# Identification and Characterization of *PTE-2*, a *Stowaway*-like MITE Activated in Transgenic Chinese Cabbage Lines

**DOI:** 10.3390/genes13071222

**Published:** 2022-07-08

**Authors:** Young-Ji Jeon, Yun-Hee Shin, Su-Jeong Cheon, Young-Doo Park

**Affiliations:** Department of Horticultural Biotechnology, Kyung Hee University, 1732 Deogyoung-daero, Giheung-gu, Yongin-si 17104, Gyeonggi-do, Korea; qdudwlq@naver.com (Y.-J.J.); yunhee94@naver.com (Y.-H.S.); so_jg@naver.com (S.-J.C.)

**Keywords:** *Brassica rapa*, transposable elements, miniature inverted-repeat transposable elements, next-generation sequencing, *Stowaway*-like family

## Abstract

Transposable elements (TEs) are DNA fragments that can be replicated or transposed within a genome. TEs make up a high proportion of the plant genome and contribute to genetic diversity and evolution, affecting genome structure or gene activity. Miniature inverted-repeat transposable elements (MITEs) are short, non-autonomous class II DNA transposable elements. MITEs have specific sequences, target site duplications (TSDs), and terminal inverted repeats(TIRs), which are characteristics of the classification of MITE families. In this study, a *Stowaway*-like MITE, *PTE-2*, was activated in transgenic Chinese cabbage lines. *PTE-2* was revealed by in silico analysis as the putative activated element in transgenic Chinese cabbage lines. To verify the in silico analysis data, MITE insertion polymorphism (MIP) PCR was conducted and *PTE-2* was confirmed to be activated in transgenic Chinese cabbage lines. The activation tendency of the copy elements of *PTE-2* at different loci was also analyzed and only one more element was activated in the transgenic Chinese cabbage lines. Analyzing the sequence of MIP PCR products, the TSD sequence and TIR motif of *PTE-2* were identified and matched to the characteristics of the *Stowaway*-like MITE family. In addition, the flanking region of *PTE-2* was modified when *PTE-2* was activated.

## 1. Introduction

Transposable elements(TEs) are genetic components that can be replicated or transposed within a genome [1]. TEs can be transposed by transposase (TPase) or replicated by reverse transcriptase (RT), which is encoded in autonomous TEs. Although non-autonomous TEs do not encode TPase or RT, it depends on the enzymes produced by autonomous TEs [2]. When autonomous TEs are excised, its derivatives arise from abortive gap repair at the excision site [2]. If the derivatives have identical terminal sequences to the original autonomous TEs, they can be recognized and activated by the TPase produced from the autonomous TEs. Derivatives with a terminal inverted repeats (TIRs) motif and target site duplications (TSDs), without any coding domains, are called miniature inverted-repeat transposable elements (MITEs). MITEs are non-autonomous class II DNA TEs. They are short (<800 bp), AT-rich, and present in high copy numbers in eukaryotic genomes [3].

Wicker et al. [4] proposed a hierarchical classification system for TEs. According to this classification system, superfamilies are distinguished by the structure of their coding proteins. In plants, six superfamilies of MITEs have been reported and abbreviated as three-letter codes: *Tc1/mariner* (DTT), *PIF/Harbinger* (DTH), *hAT* (DTA), *Mutator* (DTM), *CACTA* (DTC), and *P element* (DTP) [4,5]. Each superfamily contains several families distinguished by their conserved DNA sequences. As MITEs do not encode TPase or other proteins, it is difficult to classify MITEs by their protein domains. However, MITEs are classified by sequence homology compared with autonomous TEs. In particular, the TIR motif and TSD sequence are important for classifying MITEs. Two main families of MITEs are major in plants: *Tourist*-like family and *Stowaway*-like family. *Tourist* and *Stowaway* were the first MITEs elements discovered [6,7]. The *Tourist*-like family has target site preferences of 3 bp, TAA or TTA, whereas the *Stowaway*-like family has TA as the TSD. The autonomous superfamily encoding the TPase for the transposition of the *Tourist*-like and *Stowaway*-like MITEs are the *PIF/Harbinger* superfamily and *Tc1/mariner* superfamily, respectively, which share the same TSD sequence and TIR motif as the MITEs [8]. The other family, the *hAT* superfamily, also has the unique features of an 8 bp TSD sequence and a short TIR motif [9].

TEs are activated or repressed under stress conditions [10]. In stress conditions, plant tissue culture is the acknowledged motive for TEs activation. In rice, the retrotransposon *Tos17* has been cultured for a longer period in vitro, and a high frequency of its activation has been detected [11]. Several TEs in diverse plants have also been activated under tissue culture, such as the *Spm* and *TCUP* elements in maize [12,13] and *Tto1*, *Tto2*, and *Tnt1* elements in tobacco [14]. Plant tissue culture forms the basis of biotechnology, such as genetic transformation and subsequent regeneration. There are few studies on the activation of TEs during genetic transformation. *Tag1* and *Tos17* are the retrotransposons in Arabidopsis and rice, respectively. Both showed a higher transposition activation ratio in transgenic plants than in regenerated plants [15,16]. In Chinese cabbage (also called kimchi cabbage), a MITE named *PTE-1* was found to be activated by the transformation procedure [17]. In rice, *mPing,* a MITE in the rice genome, was mobilized in transgenic rice plants [18].

Using the resequencing data of pseudomolecule of ‘CT001’ and Chinese cabbage transgenic lines, a *Stowaway*-like MITE named *PTE-2* was characterized in this study.

## 2. Materials and Methods

### 2.1. Plant Materials

*Brassica rapa* (*B. rapa*) L. ssp. *pekinensis*, inbred line ‘CT001’ was used to develop transgenic Chinese cabbage lines. The ‘CT001’ inbred line was used as the control line for comparison with transgenic lines.

Five transgenic Chinese cabbage lines were developed from ‘CT001’ using *Agrobacterium*-mediated transformation. ‘IGA’; the *Glutathione-S-transferase* (GST) gene down-regulated transgenic lines [19], ‘COPB2’; the *Tetranychus urticae*-resistant transgenic lines [20], ‘BTTP’; the *Bacillus thuringiensis* (Bt)-resistant transgenic lines with transit peptide, ‘PPi’; the self-incompatibility down-regulated lines [21], and ‘BT’; the other Bt-resistant transgenic lines were analyzed for transposition and characteristics of MITE.

### 2.2. Identification of Activated PTE-2 by In Silico Analysis

**Figure 1:**
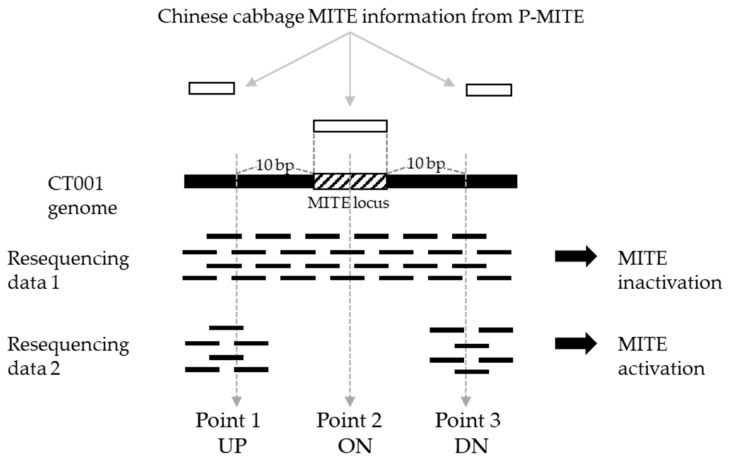
Confirmation of *PTE-2* excision by in silico analysis. UP, 10 bp upstream from *PTE-2* start locus; ON, middle of *PTE-2* locus; DN, 10 bp downstream from *PTE-2* end locus.

To detect activated MITEs in transgenic Chinese cabbage lines, a three-point analysis was conducted in a previous study [17]. The resequencing data of ‘IGA’ transgenic lines were aligned and compared with MITE-mapped ‘CT001’ pseudomolecule. The read depths at three points of the resequencing data, 10 bp upstream from *PTE-2* start locus, the middle of *PTE-2* locus, and 10 bp downstream from *PTE-2* end locus, were measured. These three points were termed UP, ON, and DN, respectively (Figure 1).

### 2.3. MITE Insertion Polymorphism (MIP) PCR Analysis

**Figure 2:**
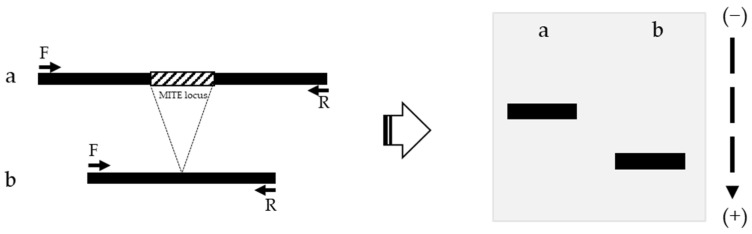
Identification of MITE activation polymorphism using MIP PCR analysis. a, MITE inactivated amplicon; b, MITE activated amplicon; F, Forward primer; R, Reverse primer.

Genomic DNA of Chinese cabbage was extracted from leaves using a RICE buffer [500 mM NaCl, 100 mM Tris-HCl (pH 8.0), 50 mM EDTA, and 1.25% (*w*/*v*) SDS]. MIP PCR analysis was used to detect MITEs transposition status (Figure 2). Forward and reverse primers were designed from 200 to 300 bp regions flanking the MITE locus. If MITE is activated, the length of the amplicon should be shorter than that of the inactivated amplicon. Primer sequences were obtained from previous research: 5′-TAT ACA TGA CGA GTA TAC GAG GG-3′ as the forward primer and 5′-CCA CAA GTG ATC GTT GTC TAG-3′ as the reverse primer [17]. BioFACT™ 2X Taq PCR Pre-Mix (BIOFACT, Seoul, Korea) was used to carry out PCR amplification with 10 pmol of forward primer, 10 pmol of reverse primer, and 50–100 ng of gDNA template. PCR amplification was conducted using a thermocycler (Applied Biosystem, Carlsbad, CA, USA) with an amplification program comprising a pre-denaturation step at 95 °C for 2 min, 35 cycles (denaturation step at 95 °C for 20 s, annealing step at 61 °C for 30 s, and extension step at 72 °C for 1 min) and a final extension step at 72 °C for 5 min.

### 2.4. Detection of the Other PTE-2 Elements at Different Loci

*PTE-2* and copy elements at different loci within the Chinese cabbage genome were investigated from the MITE-mapped pseudomolecule using the genome browser of ‘CT001’ (DNAcare, Seoul, Korea). MIP PCR was conducted for each locus to identify the activation of copy elements. The primer sets used for the MIP PCR of the copy elements are listed in Supplementary Table S1.

### 2.5. Confirmation of PTE-2 Structure and Classification

*PTE-2* and its flanking regions were sequenced from the amplicons by MIP PCR. The target amplicon was eluted from the loaded MIP PCR amplicon on a 1% agarose gel using NucleoSpin Gel and a PCR Clean-up Kit (MACHENERY-NAGEL, Duren, Germany). To obtain an accurate consensus sequence for MITEs, the eluted DNA fragment of interest was cloned into the TOPO vector using the MG TA TOPO Cloning kit (MGmed, Seoul, Korea). The consensus sequence was confirmed by multiple alignments created using CLC Sequence Viewer 8.0 (QIAGEN, Hilden, Germany). *PTE-2* structure was visualized using the mfold web server (http://www.unafold.org/mfold/applications/dna-folding-form.php/; accessed on 20 May 2020), which provides a predictive secondary structure using FASTA format.

## 3. Results

### 3.1. In Silico Detection of Activated MITE with NGS Analysis

P-MITE is a database of putative plant MITE information constructed using MITE investigation programs, MITE Digger, MITE-Hunter, and RSPB [5,22,23,24]. MITE information of Chinese cabbage from P-MITE was mapped on a pseudomolecule of ‘CT001’. It was discovered that 280,501 MITEs from the P-MITE database are distributed in the ‘CT001’ genome [17].

Among the MITE mapped on ‘CT001’, SQ041022219 was selected to be activated at the locus of the ‘IGA’ 6 transgenic lines. SQ041022219 from P-MITE was designated *PTE-2*.

### 3.2. MIP PCR Analysis of PTE-2 in Transgenic Chinese Cabbage Lines

**Figure 3:**
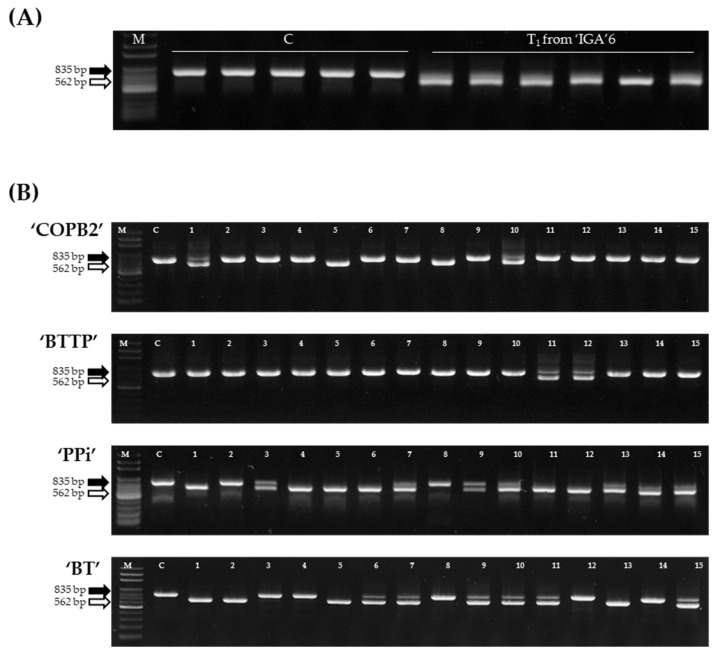
The activation analysis of the *PTE-2* by MIP PCR analysis. (**A**) MIP PCR analysis of the *PTE-2* in the control line, ‘CT001’ and the ‘IGA’ transgenic lines. M, 100 bp DNA ladder; C, ‘CT001’; lane, ‘IGA’ transgenic lines of the T_1_ generation advanced from ‘IGA’6. (**B**) MIP PCR analysis of the *PTE-2* in the control line, ‘CT001’ and four transgenic lines (‘COPB2’, ‘BTTP’, ‘PPi’, and ‘BT’). M, 100 bp DNA ladder; C, ‘CT001’; Lane, the transgenic lines. Black arrow, *PTE-2*-inserted amplicon; White arrow, *PTE-2*-excised amplicon.

To verify the in silico analysis data of activated MITEs in ‘IGA’ transgenic lines, MIP PCR was performed with advanced generations of ‘IGA’ lines. *PTE-2* loci, which were excised from ‘IGA’ 6 resequencing data, showed an activated status in the MIP PCR test of the T_1_ ‘IGA’ 6 lines (Figure 3A). These results suggested that the selection of activated MITEs by in silico analysis was valid.

Each of the 15 plants from the ‘COPB2’, ‘BTTP’, ‘PPi’, and ‘BT’ transgenic lines was used to analyze *PTE-2* activation. *PTE-2* was activated in several transgenic plants of four transgenic lines (Figure 3B). The length of *PTE-2*-excised MIP PCR products was longer than that expected in the in silico analysis (Table 1 and Figure 3).

TEs absence detection modules combined mapping and read depth coverage information to identify reads providing evidence for the presence and for the absence, respectively.

### 3.3. MIP PCR Analysis of PTE-2 Copy Elements at Different Loci in Transgenic Chinese Cabbage Lines

**Figure 4:**
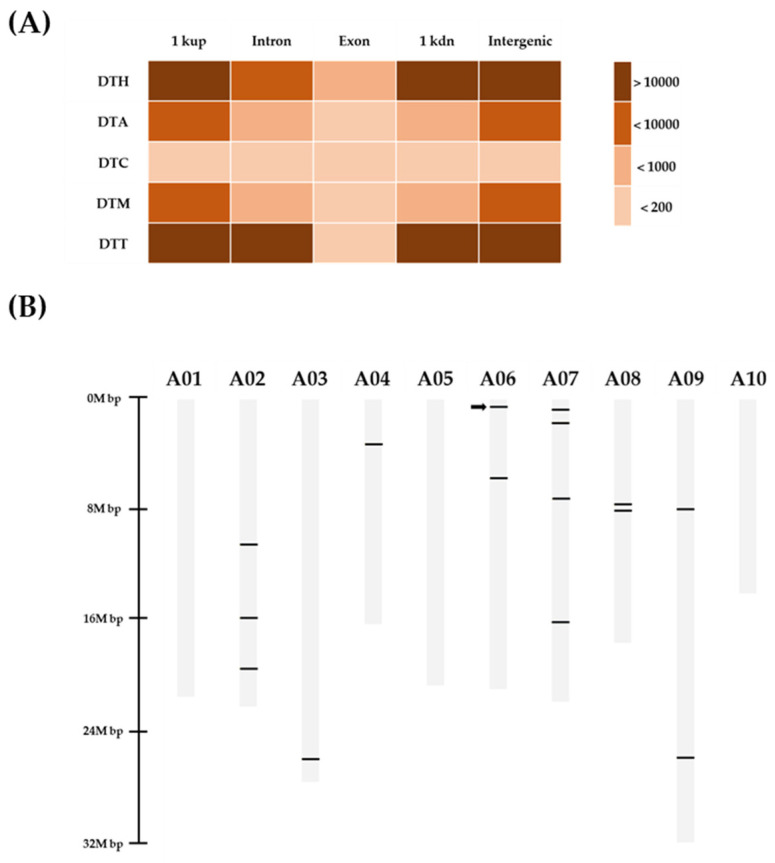
In silico mapping of *PTE-2* and copy elements in ‘CT001’ genome. (**A**) Distribution of MITE family in ‘CT001’ genome. DTT, *Tc1/mariner*; DTH, *PIF/Harbinger*; DTA, *hAT*; DTM, *Mutator*; DTC, *CACTA*; and DTP, *P element*. 1kup, 1k bp upstream from gene locus; 1kdn, 1k bp downstream from gene locus. (**B**) Location of *PTE-2* and copy elements in ‘CT001’ genome. Black bar, *PTE-2* and copy elements; Black arrow, *PTE-2* position.

MITEs in the ‘CT001’ genome were classified by family and the location of each MITE was analyzed. MITEs located in the intergenic region accounted for the largest portion, whereas MITEs located in the exonic region accounted for only a few (Table S2 and Figure 4A). The copy elements that concluded the homologous sequences of *PTE-2* were detected by the BLAST tool using the *PTE-2* sequence as a query. Fifteen *PTE-2* copy elements were distributed in the ‘CT001’ genome (Table 2 and Figure 4B). Based on in silico analysis, *PTE-2* copy elements were mapped to the ‘CT001’ genome and distributed, except for the A01, A05, and A10 chromosomes (Figure 4B). Fifteen copies (3, 1, 1, 2, 4, 2, and 2 on chromosomes 2–4, 6–9, respectively) were positioned.

To analyze the transposition activity of *PTE-2* copy elements, MIP PCR analysis of the copy elements was conducted. *PTE-2* copy elements were selected to include all loci on every chromosome and were located adjacent to the genic region. The copy elements were named *PTE-2*_cN, where N is the consecutive number (Table 2).

**Figure 5:**
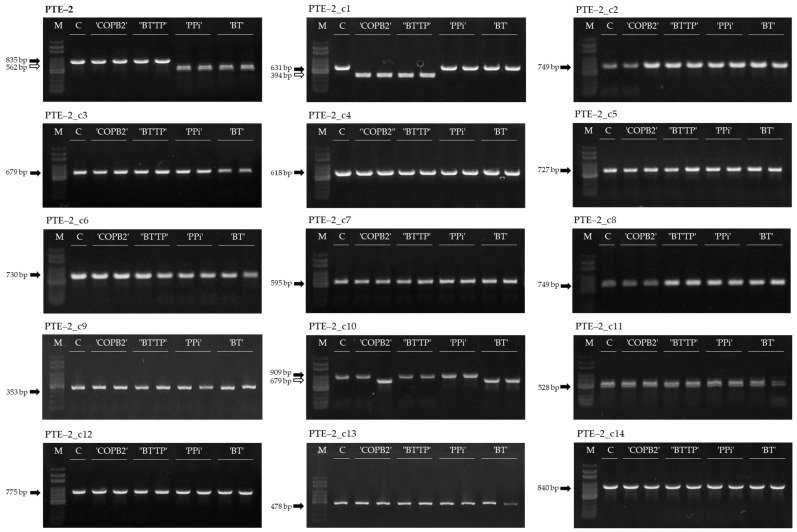
The activation analysis of *PTE-2* and copy elements at different loci by MIP PCR analysis. M, 100 bp DNA ladder; C, ‘CT001’; ‘COPB2’, ‘COPB2’ transgenic lines; ‘BTTP’, ‘BTTP’ transgenic lines; ‘BT’, ‘BT’ transgenic lines; ‘PPi’, ‘PPi’ transgenic lines. Black arrow, MITE-inserted amplicon; White arrow, MITE-excised amplicon.

MIP PCR analysis was conducted on transgenic lines that were identified to be activated. The PCR results of copy elements were compared to the loci of *PTE-2* activated in the transgenic lines (Figure 5). Among the *PTE-2* copy elements, *PTE-2*_c1 was activated in ‘COPB2’ and ‘BTTP’ transgenic lines and *PTE-2*_c10 was activated in ‘COPB2’ and ‘BT’ transgenic lines (Figure 5). In conclusion, although the copy elements had identical sequences, only a few copies were activated, and the activation tendencies were different.

### 3.4. Structural Characterization of PTE-2

To identify the *PTE-2* activated in the transgenic lines, MIP PCR amplicons were sequenced. The sequences of the ‘CT001’ and *PTE-2*-activated transgenic lines were compared to identify the *PTE-2* sequence. The *PTE-2*-inserted sequences were collected from more than three amplicons of the ‘CT001’ lines to obtain the *PTE-2* consensus sequence. The *PTE-2* consensus sequence was used to confirm structural characteristics. The secondary structure of *PTE-2* was displayed using the mfold web server (http://www.unafold.org/mfold/applications/dna-folding-form.php/; accessed on 20 May 2020), which provides a predictive secondary structure from the sequence of nucleic acids [25]. The TIR motif was investigated from the *PTE-2* secondary structure, which analyzes hairpin-like base pairing with the 5′ and 3′ ends of *PTE-2*. The TSD sequence was investigated by comparing the excision site of *PTE-2*-excised transgenic lines with that of the *PTE-2*-inserted ‘CT001’ lines. The TSD sequence was duplicated and flanked by the *PTE-2* sequence at the *PTE-2*-inserted sequence, whereas the single TSD sequence remained at the *PTE-2*-excised sequence. The superfamily of the *PTE-2* was classified based on its TSD sequence and TIR motif. The CENSOR web server (https://www.girinst.org/censor/; accessed on 9 June 2020) was used to confirm the similarity to previously described repetitive DNA sequences collected in the Repbase database [26].

**Figure 6:**
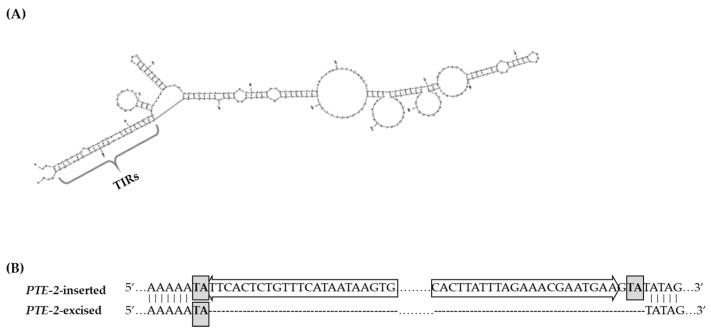
Confirmation of structural characteristics of the *PTE-2*. (**A**) A secondary structure was constructed from consensus sequence of the *PTE-2*. The entropy values (ΔG) indicating the stability of this secondary structure was −19.16. The brace indicated the base pair produced by the TIR motif. The TIR motif was determined by comparing the 5′ terminal sequence and reverse-complementary sequence of 3′ terminal. (**B**) The remaining TSD sequence from excision of the *PTE-2*. Grey box, TSD sequence; White arrow, TIR motif.

The *PTE-2* had 78% of A+T content and was 268 bp in length. The TSD sequence of *PTE-2* was 5’–TA–3’, which is homologous to the *Stowaway* MITE family (Figure 6B). The TIR motif of *PTE-2* was determined to be 23 bp, with base pairing in the secondary structure (Figure 6A). Using the consensus sequence of the *PTE-2* as a query for the CENSOR tool, it was masked to the *Tc1/Mariner* DNA transposon in the *Brassica oleracea* genome, matching the 2 bp TSD sequence and the 26 bp TIR motif (Table 3).

### 3.5. Flanking Sequence Duplication of PTE-2-Excised Site

**Figure 7:**
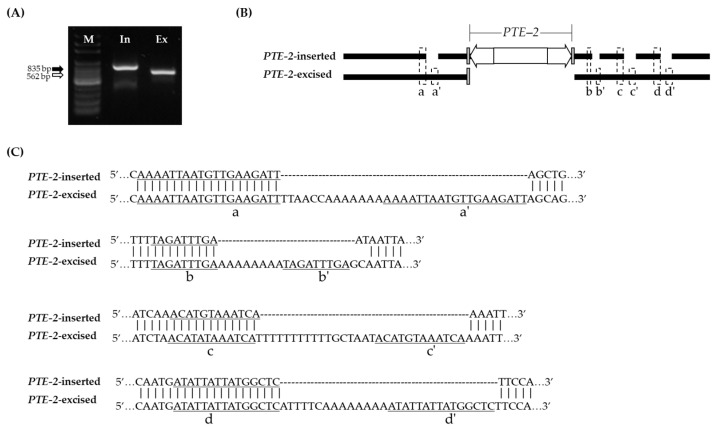
Comparison of *PTE-2*-inserted and -excised sequence. (**A**) MIP PCR analysis of *PTE-2*. M, 100 bp DNA ladder; In, *PTE-2* inserted sequence amplicon; Ex, *PTE-2* excised sequence amplicon. (**B**) Alignment of *PTE-2* inserted sequence and excised sequence. Grey box, TSD sequence; White arrows, TIR motif. (**C**) Four duplicated regions that were flanking to *PTE-2* excised site. a’, b’, c’, and d’ indicate the sequences that were duplicated from a, b, c, and d, respectively, when the *PTE-2* had been activated.

The product of the MIP PCR analysis with the *PTE-2*-excised fragment was identified to be longer than expected (Figure 7A). The amplicons were sequenced to align them with the *PTE-2*-inserted sequences. As a result, there were four nucleotide insertions adjacent to the *PTE-2*-excised site, which were generated by duplication of the *PTE-2* flanking sequence (Figure 7B). The insertions were generated by duplications of the sequences before each insertion (Figure 7B,C). In Figure 7C, *a’* is a duplicated sequence from the *a* sequence and it was 19 bp in length. Between *a* and *a’*, the 13 bp sequences, including (A)_7_ microsatellites, were spaced. *b’* is a duplicated sequence from the *b* sequence and it was 9 bp in length. Only eight microsatellites were spaced between the *b* and *b’*. c’ is a duplicated sequence from the *c* sequence and it is 12 bp in length. Between *c* and *c’*, 17 bp of sequences, including (T)_11_ microsatellites, were spaced. *d’* is a duplicated sequence from the *d* sequence, and it was 15 bp in length. Between *d* and *d’*, 14 bp of sequences, including (A)_8_ microsatellites, were spaced.

## 4. Discussion

The number of studies on TEs has increased for many crops. McClintock observed that the specific locus on chromosome 9 of maize had broken frequently and termed this locus a dissociator (*Ds*) element. The *Ds* element can move to a new location within the genome only if the activator (*Ac*) element has provided a transposase (TPase), which is responsible for the transposition event of *Ds* element [27]. The *Ac*/*Ds* system represents the relationship between autonomous TEs and non-autonomous TEs. TEs can be divided into two major classes. Class I retrotransposons possess a copy-and-paste transposition mechanism. The class I element is transcribed into mRNA, the intermediate of its transposition, by RNA polymerase II. The mRNA was converted into to cDNA by RT and integrated into the new location of the genome. Because of its transposition event, the retrotransposon element is replicated to a new location, remaining in the donor location. In contrast, class II DNA transposons exhibit a cut-and-paste mechanism. Class II elements are excised from their donor location and moved to a new location by TPase [28]. Although DNA transposons are not replicated through the transposition process, *PTE-2*, a class II element, has multiple copies within the genome (Figure 4). MITEs are the major type of TEs, comprising approximately 4.05% of the Chinese cabbage genome by having 6637 different elements and 280,501 copies [17].

TEs make up a high proportion of the plant genome, accounting for 18.5% of *Arabidopsis thaliana*, 58.7% of *Glycine max*, 39.5% of *Oryza sativa* (*O*. *sativa*), and 84.7% of *Zea mays* [29]. The transposition and amplification of TEs contribute to genetic diversity and evolution, affecting genome structure and the gene activity [30]. The TE-Thrust hypothesis states that TEs have the potential to facilitate evolution by promoting ectopic recombination and reformatting genomes by TE transposition and integration [31].

P-MITE is a database of MITE information for 41 plant species. A total of 174 MITE families, including 1 DTC, 11 DTM, 16 DTT, 56 DTH, and 90 DTA families, in the *B. rapa* reference genome were published in the P-MITE database [5,32]. The MITE information derived from computer programs can be used to develop MITEs characteristics and to study the dynamics of MITEs in plant genomes. From the structural characteristic analysis, *PTE-2* was classified as the DTT family, the third largest family in *B. rapa*. Because the *Tc1/Mariner* superfamily of DNA transposons was considered to be the origin of the *Stowaway*-like family via its internal deletion [33], *PTE-2* was classified into the *Stowaway*-like family based on its 2 bp TSD sequence of 5′–TA–3′ and TIR motif homology to the *Tc1/Mariner* superfamily. Although studies on the activity of MITE belonging to the DTT family are limited, studies have shown that gene expression changes according to the activity of MITE belonging to the DTM family. *MnM2*, a member of the *Mutator* family, regulates the *MnANR* gene associated with the color of tobacco flowers in mulberry trees (*Morus notabilis*). The expression level of the *MnANR* gene in transgenic plants was higher than that in the wild type [34].

TEs are activated or repressed under stress conditions [10]. In stress conditions, plant tissue culture is the acknowledged motive for MITE activation. In rice, *mPing*, a MITE in the *O. sativa* genome, was mobilized in transgenic rice plants [18]. *nDaiZ*, a member of the *hATt* family, was activated during tissue culture. In scutellum-induced rice plants, *nDaiZ* was confirmed to transpose another genomic region through PCR analysis [35]. In addition, peanut *AhMITE1*, was activated during tissue culture. *AhMITE1* was activated with a 6.25% transposition frequency in cultivar “Tifrunner” [36]. These results indicate that tissue cultures may create an appropriate environment for transposon activation in plants.

MITEs have contributed to the evolution of the *Brassica* genome by comparing 20 MITEs that have shown dynamic activity throughout the *Brassica* genus [37]. *PTE-1*, which is a MITE in the *Brassica* genome, was found to be activated during the transformation procedure [17]. Likewise, *PTE-2*, *PTE-2_c1*, and *PTE-2_c10* were partially activated in transgenic Chinese cabbage lines (Figure 3 and Figure 5).

Activated MITEs have been used to analyze the relationship between changes in the characteristic variations in various food crops. In maize, early flowering was induced by MITE insertion in major quantitative trait loci related to flowering time [38]. The MITE insertion position was extraordinarily methylated and related to the *ZmRap2.7* transcription level. In addition, MITE inserted upstream of the multidrug and toxic compound extrusion (MATE) gene increased aluminum toxicity as the gene expression changed [39]. As MITEs were activated and inserted into the gene, color variations were observed in potato tuber skin [40] and gentian petals [41].

In general, DNA transposons left only a single TSD or footprint, the remnant sequence from the excised transposon sequence when TEs have been activated [42,43]. When *PTE-2* elements were activated, only a single TSD sequence was left at the excised site; however, the sequences flanking the *PTE-2*-excised site were modified by generating insertions in four regions. It was confirmed that nucleotides A and T remained in the *PTE-2* adjacent region when activated (Figure 7B,C). When *PTE-2* was activated, duplications and mononucleotide repeats were generated near the *PTE-2*-excised site. A few studies have analyzed the modifications induced by TEs transposition. The overall frequency of nucleotide substitutions and indels increased in the TEs-excised site [44]. TEs activation can trigger the generation of tandem repeats [45], and the genomic structure can be modified by the insertion of MITEs without TPase. Gene expression may be affected by the activation or inactivation of MITEs or by small RNA derived from MITEs [46,47].

## 5. Conclusions

TEs were composed of 40% *B. rapa* genome. In particular, MITEs, which are TEs, have high copy numbers and play a significant role in genetic evolution. In this study, *PTE-2* was selected by resequencing data from in silico analysis. MIP PCR was performed to identify MITE activation polymorphisms. The activation tendency of *PTE-2* and copy elements at different loci was confirmed in transgenic Chinese cabbage lines. Sequencing and analysis of the TSD sequence and TIR motif of *PTE-2* classified it as a *Stowaway*-like family. In addition, when *PTE-2* was activated, duplications and mononucleotide repeats were generated adjacent to the *PTE-2*-excised site. The results of this study indicated that MITEs are activated during tissue culture and transformation and will provide helpful information for the genetic diversity of the plant genome.

## 6. Patents

We are in the process of obtaining a patent for the data in Korea (patent application number 10-2021-0055780; application date 29 April 2021).

## Figures and Tables

**Table 1:** *PTE-2*-excised detection combine mapping and read depth coverage.

Line	UP ^z^	ON ^y^	DN ^x^
‘CT001’	6.3	8	6
‘IGA’6	7	0.09	3

^z^ Read depth coverage at a point 10 bp upstream from *PTE-2* start locus. ^y^ Read depth coverage at the middle of *PTE-2* locus. ^x^ The read depth coverage at the point 10 bp downstream from *PTE-2* end locus.

**Table 2:** *PTE-2* copy elements at different loci in ‘CT001’ genome.

cN ^z^	Chr ^y^	Start	End	Strand	Length (bp)	Identity ^x^
*PTE-2*	A06	637,582	637,854	+	273	100
*PTE-2*_c1	A02	10,813,956	10,814,192	+	238	86.97
*PTE-2*_c2	A02	16,240,447	16,240,682	+	237	86.08
*PTE-2*_c3	A02	19,985,345	19,985,574	+	239	86.19
*PTE-2*_c4	A03	26,668,867	26,669,128	-	278	82.01
*PTE-2*_c5	A04	3,356,039	3,356,288	-	252	84.92
*PTE-2*_c6	A06	5,906,593	5,906,822	+	233	87.12
*PTE-2*_c7	A07	814,580	814,816	+	240	80.42
*PTE-2*_c8	A07	1,776,244	1,776,481	+	239	88.28
*PTE-2*_c9	A07	7,412,914	7,413,154	+	244	86.07
*PTE-2*_c10	A07	16,535,966	16,536,195	+	236	80.51
*PTE-2*_c11	A08	7,875,571	7,875,815	-	248	86.69
*PTE-2*_c12	A08	8,011,522	8,011,748	-	229	87.77
*PTE-2*_c13	A09	8,224,067	8,224,306	-	244	88.53
*PTE-2*_c14	A09	26,516,545	26,516,815	+	280	82.50

^z^ *PTE-2* copy elements, where N is the consecutive number. ^y^ MITEs loci on ‘CT001’ chromosome. Chr, chromosome. ^x^ The identity value was derived by a BLAST search using *PTE-2* sequence as a query.

**Table 3:** The structural characteristics of *PTE-2*.

A + T (%)	Length (bp)	TSD (5′–3′)	TIR (5′–3′)	MITE Family
78	268	TA	TTCANTCTGTTTCNNAATAAGTG	*Stowaway* (DTT)

## Data Availability

Not applicable.

## Supplementary Materials

The following supporting information can be downloaded at https://www.mdpi.com/article/10.3390/genes13071222/s1, Table S1: List of primer sets for MITE insertion polymorphism (MIP) PCR analysis, Table S2: Distribution of MITE family in “CT001” genome.
