# Supplementary material for: Identification and Characterization of PTE-2, a Stowaway-like MITE Activated in Transgenic Chinese Cabbage Lines
Source: Genes (Basel). 2022 Jul 8;13(7):1222. doi: 10.3390/genes13071222 (PMC9319602; doi:10.3390/genes13071222)
Supplement: Supplementary file 1 [file genes-13-01222-s001.zip › genes-1810197-supplementary.pdf]

Table S1. List of primer sets for MITE insertion polymorphism (MIP) PCR analysis.

MITE name	Primer name	Primer sequence (5'→3')	Inserted size (bp)	Excised size (bp)
PTE-2	*PTE-2* F[Z]	TATACATGACGAGTATACGAGGG	835	562
	PTE-2 R	CCACAAGTGATCGTTGTCTAG		
*PTE-2*_c1	*PTE-2*_c1 F	CATGTAGATTTACCCCAACG	631	394
	*PTE-2*_c1 R	CGCCTAGTCTCAACTTCAAA		
*PTE-2*_c2	*PTE-2*_c2 F	CACGATGATGGCGAGATTATT	749	513
	*PTE-2*_c2 R	TGTTGAACTATGACCGAGTGG		
*PTE-2*_c3	*PTE-2*_c3 F	TTGGTATGCATGAATGGCAC	679	449
	*PTE-2*_c3 R	GGTACCTGCTTGGTGTACATT		
*PTE-2*_c4	*PTE-2*_c4 F	ATTGTACCAGAAAGATGTATATT	618	356
	*PTE-2*_c4 R	GTCAAGTTCATTTACATACTC		
*PTE-2*_c5	*PTE-2*_c5 F	GCCACACGAGCCTGATGC	727	477
	*PTE-2*_c5 R	GCGGTGATGTTTAATCCAGCG		
*PTE-2*_c6	*PTE-2*_c6 F	GCCCAACAACAACAATAATAAT	730	500
	*PTE-2*_c6 R	CCGAATCCAGATCCAGGT		
*PTE-2*_c7	*PTE-2*_c7 F	AGACCCTAAGTGTAATCAA	595	358
	*PTE-2*_c7 R	AAAGATAAAGATGTAGAGTTGTT		
*PTE-2*_c8	*PTE-2*_c8 F	TGCATGAATGATTATGTCGAT	749	511
	*PTE-2*_c8 R	GGAATGTGAGTAGTATGAAG		
*PTE-2*_c9	*PTE-2*_c9 F	CATCTCTATTTCTCTATTTCCAA	353	112
	*PTE-2*_c9 R	TGGAAATAGAGGTGGAATA		
*PTE-2*_c10	*PTE-2*_c10 F	TCTCTATGAATCCCAACTTA	909	679
	*PTE-2*_c10 R	CTTTCAGTAGGGTAGCAT		
*PTE-2*_c11	*PTE-2*_c11 F	CTAATCGCCACATAAACTAACC	528	283
	*PTE-2*_c11 R	TAACACGAGAGTAGTCAATAGG		
*PTE-2*_c12	*PTE-2*_c12 F	GAAACCAGAGAACACCATAC	775	548
	*PTE-2*_c12 R	TGTGAAATGTTCGGTTACTCTT		
*PTE-2*_c13	*PTE-2*_c13 F	TTC AAT AGT AGT TCA CCG	478	238
	*PTE-2*_c13 R	TTC ATC TTG TAA TCT GTA AT		
*PTE-2*_c14	*PTE-2*_c14 F	CTCCTACTTCATCTCTTCAAG	840	569
	*PTE-2*_c14 R	CTAACTTCTGTTACAGTCAATG		

[Z]F, forward primer; R, reverse primer

Table S2. Distribution of MITE family in 'CT001' genome.

	1kup[z]	Intron	Exon	1kdn[y]	Intergenic	Total
DTH	28,546	9,159	808	18,945	67,419	124,877
DTA	1,269	564	44	735	2,847	5,459
DTC	6	6	0	17	96	125
DTM	1,798	426	7	765	2,041	5,037
DTT	35,682	13,859	114	22,786	72,562	145,003

[z]: 1kup, 1k bp upstream from gene locus. [y]:1kdn, 1k bp downstream from gene locus.
